# HCV–HIV Coinfection: Simple Messages from a Complex Disease

**DOI:** 10.1371/journal.pmed.0040240

**Published:** 2007-10-09

**Authors:** Paul Klenerman, Arthur Kim

## Abstract

The authors discuss our current understanding of the immunopathogenesis of HCV-HIV coinfection.

Hepatitis C virus (HCV) and HIV infection are both major global health problems, each with their own specific unsolved and difficult issues of prevention, pathogenesis, and therapy. For HIV, many of the clinical problems experienced are related to loss of immunological control over relatively commonly encountered pathogens. In most of these cases (e.g., cytomegalovirus [CMV], Pneumocystis jiroveci. human herpesvirus-8), normal immunological control is quite efficient, and these organisms behave as “opportunists.” HCV is slightly different, in that immunological control in normal HIV-uninfected individuals is often poor, and HCV infection alone can lead to the gradual evolution of end-stage liver disease in normal hosts. However, although a consensus is forming about the basic details of the immune responses associated with acute control of HCV monoinfection, the long-term relationships between immune responses, viral load, and most importantly, disease progression in those who are persistently infected are still poorly understood.

HIV–HCV coinfection is a problem of substantial size. Overall the figures for coinfection rates are very striking—around one in 10 of those infected with HIV globally are also HCV infected (around 4–5 million) [1]. In the West, however, the infection rate is one in four, and in specific risk groups, such as intravenous drug users, the figure rises to up to 50%–95%, regardless of global location. This reflects the fact that HCV is relatively easy to spread through percutaneous infection, while relatively harder to spread through sexual contact.

The coinfection rate would matter little on its own if the consequences were not so grave. HIV infection has a major effect on HCV viral load [2] and a clinically significant effect on HCV disease progression [3]. Furthermore, although effective antiretroviral therapy (ART) has transformed the course of HIV disease progression, HCV-related liver disease is still a significant cause of morbidity and mortality in this group [4].

In this Research in Translation article, we discuss our current understanding of the immunopathogenesis of HCV–HIV coinfection. We firstly discuss what is known of the host immune response in HCV monoinfection, and subsequently the impact of HIV on this response. We focus on two issues—immune deficiency and immune dysregulation, which we propose combine to produce the clinical picture associated with coinfection.

## Immune Responses during HCV Infection

### Resolving infection.

HCV sets up long-term persistence in the majority of those infected—around 75% overall [5]. In individual cohorts, however, the spontaneous clearance rate may be somewhat higher, up to 50% [6]. The mechanisms that contribute to spontaneous clearance include innate and adaptive immunity. At the time of acute infection, there is evidence for the generation of strong T cell responses, both CD4^+^ and CD8^+^, which are temporally associated with control of viremia [7,8]. Furthermore, in animal model experiments, removal of these subsets leads to failure to control viremia [9,10]. At the genetic level, in human infection there is the linkage between specific human leukocyte antigen (HLA) class I and class II molecules (e.g., HLA-B27 [6] and HLA-DR11 [11]) with clearance of virus, indicating that the presentation of specific viral peptides through molecules leads to protective responses. Data illuminating the immunologic basis of these genetic associations with disease outcome are emerging [12].

Although antibody responses are mounted, these are somewhat delayed, and limited by the rapid emergence of escape mutants (see below). The emerging consensus is that strong T cell responses accompanied by innate mediators dominate to control infection successfully. If virus is contained in this acute phase, strong memory T cell responses are detectable in the long term, although antibody responses may wane relatively quickly [13].

### Persistent infection.

In those who become persistently infected, the virus must evade host innate and adaptive immunity. The fundamental differences between the initial responses that are successful in containing virus, compared to those that are unsuccessful, have been difficult to pinpoint. Most individuals do mount measurable CD4^+^ and CD8^+^ T cell responses in the acute phase, but if virus persists, these responses are generally not sustained at high levels [8,14]. Many responses become undetectable by conventional assays, or are present at very low levels in blood. Those that are detectable have been described as weak in specific functions, notably proliferative capacity [15]. This picture contrasts strongly with other persistent virus infections such as CMV or HIV, although similar loss of reactivity is seen in chronic hepatitis B infection [16].

Evasion of host adaptive immunity by HCV and downregulation of T cell responses may occur through a number of mechanisms. Firstly, the high levels of viral production (greater than 10^10^ viral particles per day) and the variable viral genome leads to emergence of escape mutations. These are evident both in the envelope genes, as a consequence of antibody-mediated selection pressure [17], and throughout the genome within specific CD8^+^ T cell epitopes [18,19]. The capacity of HCV to escape both antibody and T cell responses is evident in longitudinal studies of infected individuals, but also to some extent in cross-sectional analyses of infected patients, where the “footprint” of specific HLA-restricted T cell responses may be observed [19,20].

A further mechanism that may help HCV elude host immunity is the induction of T cell exhaustion. This phenomenon relates to the sequential loss of T cell functions in the presence of high levels of virus, followed ultimately by the deletion of such cells. The mechanisms leading to exhaustion are not fully clear. One line of recent evidence suggests that the inhibitory molecule programmed death-1 may be implicated [21]. A similar mechanism probably occurs in both CD4^+^ and CD8^+^ T cells and has been demonstrated in chronic HIV infection [22]. Exhaustion of CD8^+^ T cells is strongly influenced by the function of CD4^+^ T cells in animal models [23]—thus loss of CD4^+^ T cell activity profoundly affects the ability to sustain functional antiviral CD8^+^ T cell populations.

A final factor that is hypothesized to modify the host T cell response to HCV is the induction of regulatory T cell subsets. Such cells, which are best tracked using the expression of transcription factor FOXP3, have been demonstrated in many chronic inflammatory states and infections, including HCV [24]. Regulatory T cells may be generated in the thymus (natural Tregs) or in the periphery, as a result of antigenic stimulation, and act to inhibit function of antigen-specific T cells, including HCV-specific CD8^+^ T cells [25,26]. They may produce interleukin-10 (IL-10) and transforming growth factor-beta (TGF-beta), both of which are inhibitory cytokines. It is generally considered that such T cell populations serve to limit immunopathology and autoimmunity. Further careful study is needed in this arena to examine the role of these cells in HCV infection.

Multiple studies using diverse techniques demonstrate that patients who present with chronic HCV infection exhibit relatively weak ex vivo T cell responses in blood assays (reviewed in [27]). It has been very difficult to relate these responses closely to either the viral load or the degree of liver inflammation and/or hepatic fibrosis. This may be due in part to the technical difficulties of such studies—it has generally been hard to define such relationships in HIV, which elicits much larger T cell responses and has been much more extensively investigated (reviewed in [16]). It is also difficult to correlate measurements from one time point at liver biopsy with an outcome (fibrosis) that often takes decades to develop. In addition, the enormous antigenic variation present in HCV makes such assays difficult both to perform and interpret. Further complications emerge from the failure to adequately visualize responses in the liver, the main focus of the pathology, as will be discussed below.

## Immune Responses in the Liver

A number of studies have attempted to analyze CD8^+^ and CD4^+^ responses in the liver of HCV-monoinfected patients. This is rarely done in acute disease outside animal model settings. When studied acutely, there is a substantial period where virus is present at high levels, but no tissue damage or inflammatory infiltrate is observed [9,10]. This is important for two reasons. Firstly, it suggests that on its own, the virus causes relatively little tissue damage, at least in the short term, and thus explains the lack of a robust link between viral load and disease activity (see below). Secondly, the severe delay in mounting an adaptive immune response (often several weeks), despite the presence of replicating virus, is probably central to the capacity of HCV to set up long-term persistence, since it has already established an important numerical advantage [28].

The liver is a site of enrichment for memory T cell populations, regardless of the virus, as has been shown in well-controlled murine models [29]. Similarly, enriched populations of CD8^+^ T cells specific for Epstein-Barr virus and CMV may be found even in normal human liver [30]. Chronic HCV infection studies using major histocompatibility complex class I peptide tetramers (which can directly identify antigen-specific T cells, regardless of their function) have also shown relatively enriched populations in liver biopsies compared to blood [31,32]. In some cases, T cell populations may fall below the level of detection in blood but be demonstrated in the liver. However, generally such populations are still less than 1% to 20% of the total CD8 infiltrate—i.e., the majority of the T cell infiltrate in the liver in chronic HCV is not apparently HCV specific.

It also appears that the CD8^+^ T cells in the liver may have an unusual cytokine secretion profile, with some studies linking them with IL-10 secretion [33,34]. Antiviral CD8^+^ T cells typically secrete interferon (IFN)-gamma, and this has also been shown in T cells derived from the liver. However, a relative increase in IL-10 secretion within liver tissue has been proposed to limit the tissue damage associated with these T cell infiltrates, and an inverse relationship with hepatic inflammation has been shown [33]. Relatively little is known about the quality and quantity of virus-specific CD4^+^ T cells in the liver, although IL-10 secretion in this subset has been reported [35]. A recent study has shown that around 40% of the CD4^+^ infiltrate in HCV-monoinfected livers is FOXP3+, regardless of histologic stage [36]. This was much higher than the figure in autoimmune liver disease, and implies a very large hepatic regulatory T cell presence in chronic HCV.

**Five Key Studies in the Field**

1. **Cox et al., 2005** [18] This paper revealed the extent of immune escape from CD8^+^ T cells that occurs after acute HCV infection.
2. **Kim et al., 2005** [56] This paper was the first to look comprehensively at HCV-specific CD8^+^ T cell responses in coinfected patients, and revealed a deficit associated with lowered CD4 counts.
3. **Accapezzato et al., 2004** [33] This paper provided evidence for an unexpected regulatory role of CD8^+^ T cells in the liver in HCV.
4. **Grakoui et al., 2003** [9] This paper showed clear evidence for a protective role of CD4^+^ T cells against HCV persistence and reinfection.
5. **Thomas et al., 2000** [39] This clinical study revealed the clear impact of HIV on control of HCV viral load in chronic infection.

These data indicate overall that in chronic HCV infection antiviral T cells do home to the infected liver and continue to secrete antiviral cytokines. However, within the hepatic environment, there appears to be a regulatory process occurring, which likely comprises multiple cell subsets. In other words, the T cell infiltrate in the liver probably contributes to three main effects: control of virus replication, induction of hepatic inflammation, and regulation of such inflammation (Figure 1).

**Figure 1:**
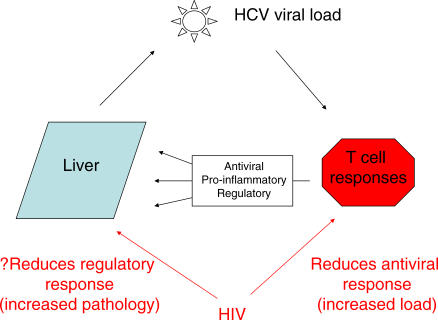
Immunologic Control of HCV Pathogenesis In a normal HCV monoinfection, HCV-specific CD4 and CD8 T cells are mobilized to contain infection. If infection is not cleared, such T cells contribute to antiviral effects, proinflammatory effects, and regulatory effects. There is emerging evidence that the high viral loads of HCV during coinfection with HIV are associated with profound defects in HCV-specific CD4 and also CD8 T cell responses. There are some data showing that the cytokine environment in the liver is altered, which could affect disease progression independently from any influence of virus load.

## The Main Clinical Effects of HIV Infection

HIV coinfection significantly affects every stage of HCV disease progression (Table 1). Firstly, a higher rate of viral persistence is seen [2,37]. Secondly, and most strikingly, higher viral loads—about 0.5 to 1 log higher on average than in monoinfected individuals—are seen in most studies [2,38,39]. This high load is associated with higher transmission rates, for example in mother-to-child transmission studies [40]. It may also contribute to the recent outbreaks of transmission of HCV amongst HIV-positive men in European and North American cities [41]. The higher loads are also associated with a reduced response to therapy using standard interferon/ribavirin combinations [42].

**Table 1:**
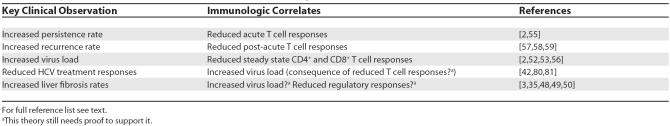
Major Clinical Impacts of HIV on HCV and Immunologic Correlates

Most important, however, as far as patients are concerned is the fact that accelerated fibrosis and rates of cirrhosis have all been observed, leading to increased morbidity and mortality associated with HCV for HIV-infected individuals [4,43–46]. This is an important finding clinically, but also perhaps an unexpected one. Many studies of HCV have suggested that disease progression is not closely linked to virus load, unlike in HIV [47]. Interestingly, there are some studies which do link HCV load and disease progression, although such cohorts may also include HIV-coinfected subjects [47,48]. Other cofactors that may accelerate the disease process in HIV–HCV coinfection are alcohol use, age, gender, and race. Confounding this further is the presence of other metabolic issues such as steatosis, which may be related to antiretroviral therapies. Despite all these cofactors, HIV-induced immunosuppression has an independent effect on fibrosis progression [49,50].

How are these effects of HIV on HCV infection explained? In particular, to what extent do the current immunological data help us understand the distinct phenomena associated with coinfection—most notably a rise in viral load and an increase in disease progression (Figure 1)?

## Impact of HIV on HCV-Specific Immune Responses

### Impact on virus load.

While HIV-mediated effects on the immune system are complex, two primary outcomes are the decline of CD4^+^ T lymphocytes, resulting in AIDS, and chronic immune activation. Shortly following acute HIV infection of humans and macaques, a massive depletion of mucosal memory CD4^+^ T cells is observed, which is only partially reversed by antiretroviral therapy [51]. Then with progressive infection, a generalized chronic immune activation state accompanies the gradual decline in immunity to various pathogens. Given both of these effects, one would predict loss of the quantity and function of HCV-specific T cells, particularly CD4^+^ T cells, in coinfected persons.

Studies of peripheral blood mononuclear cells indicate that HCV-specific CD4^+^ T cells are at extraordinarily low frequency in coinfected subjects, whether measured by proliferation or cytokine secretion [52–54], including some emerging data from acute disease [55]. This loss of antiviral CD4 responses has not been closely linked to CD4 T cell count, and may therefore occur relatively early. The observation of loss of HCV-specific CD4^+^ T cell responses is striking for two reasons. Firstly, they are already considered relatively weak responses in chronic monoinfection, by comparison with those with resolved infection, or with responses to other persistent pathogens [16]. However, it is clear that these responses may be further impaired and these defects may be linked to further loss of control over the virus. In other words, the response in persistent HCV, while ineffective at controlling the virus completely, may well be setting an important limit on virus load. What is not yet clear, however, is why HCV-specific responses should be relatively sensitive to the effects of HIV, given that responses to other infectious agents only decline substantially at later stages of HIV progression.

In addition to profound loss of virus-specific CD4^+^ T cells, there is documented failure of CD8^+^ T cell responses. Peripheral total CD4 counts correlate with the magnitude and breadth of the HCV-specific CD8 IFN-gamma response [56]. This is in stark contrast to the HIV-specific CD8 response, which can persist despite high HIV viral loads and low CD4 counts. Interestingly, in a subset of those with spontaneous control of viremia, an increased rate of viral recurrence in coinfected subjects has been seen [57–59]. One study was able to measure immune responses before and after recurrent HCV and found that preserved immunity correlated with lower viral loads following recurrence [57]. This latter observation further supports the idea that in chronic HCV monoinfection, although responses are somewhat limited by escape, exhaustion, and regulation, they nevertheless make an important contribution towards control of viremia, including during a secondary challenge with HCV, a control which is severely disrupted by the action of HIV.

### Impact on disease progression.

The consequences of depletion of IFN-gamma-secreting T cells, as detected in the periphery, upon the pathogenesis of HCV infection within the liver remain unknown. We propose three basic models which might explain this important link.

### 1. Impact through increased virus load alone.

Since HIV depletes HCV-specific T cells, and this is accompanied by an increase in virus load, the simplest model would be—as for other pathogens—that the increase in load is responsible for the disease progression (in this case, inflammation/fibrosis). The problem with this hypothesis is that in monoinfection, the link between load and pathology is not very strongly supported [47], although it has been observed [48]. It is therefore possible that the extremely high HCV loads seen in HIV–HCV coinfection, over very long periods, have additional direct and detrimental effects on the liver, or indirect effects in combination with other mechanisms [60].

### 2. Loss of regulation.

As mentioned above, HCV infection is associated with the emergence of a number of immune regulators, including FOXP3+ regulatory T cells and IL-10-secreting intrahepatic CD8^+^ T cells. The anti-inflammatory effects of these may contribute to control of liver damage in the short term and thus may be antifibrotic in the long term. While the intrahepatic environment in HCV–HIV coinfection has been the subject of only limited study, histologically there is a profound loss of CD4^+^ T cells [61]. One study demonstrated a depletion of total intrahepatic CD4 T cells out of proportion to declines in peripheral counts, indicating that the inflammatory infiltrate in the liver is missing a key component of an effective T cell response. Given that nearly half of these cells may be FOXP3+ [36]—i.e., with likely regulatory function—this could severely affect both antiviral and anti-inflammatory function. In keeping with this, functional data suggest a loss of IL-10-secreting cells [35]. The reported HIV-associated dysregulation of various cytokines [62], including TGF-beta, IL-10, and IFN-gamma, secreted by the cellular infiltrate may tilt the balance towards a profibrotic state. The regulation of liver fibrosis is complex—although it has been classically linked to Th1-mediated tissue injury, there is emerging evidence that Th2 cell subsets may play a proinflammatory role, with a particularly important role for IL-13-secreting CD4^+^ T cells [63]. Much more work is required in both mono- and coinfection to assess the role of pro- as well as anti-inflammatory subsets in this organ.

### 3. Indirect effects.

While we have focused here on the specific T cell–mediated antiviral responses, HIV and HCV both have effects on other cell subsets, including NK cells [64,65], NKT cells [65,66], gamma delta cells [60,66], and potentially on cytokine-secreting antigen-presenting cells in the liver [67]. It remains possible that the impact of HIV on disease pathogenesis occurs through modulation of such cells. B cell responses to HCV are also diminished in the presence of HIV [68]. Although all these theories are possible, it will be extremely difficult to disentangle the role of any one of them on disease progression due to the multitude of potentially interacting cell subsets, independent from the profound effects described above.

## Treatment Considerations

### HCV therapy.

The adverse impact of HIV on treatment outcome for HCV has been well documented. While there have been advances, even with the best of treatments we do not successfully treat HCV infection in the majority of those with coexisting HIV [69]. What explanations do studies of HCV immunopathogenesis offer us?

The simplest model is that virus clearance induced by interferon-based therapy is closely linked to pretreatment virus load [42]. Thus failure of the immune response to contain virus in the steady state could alone account for a failure of antiviral therapy in the long term. There has, however, been much interest in the role of immune responses in long-term treatment outcome. One reason for this is that both interferon and ribavirin have immunomodulatory roles, and thus it seems plausible that these could contribute to the treatment effect—especially since the direct antiviral effect of ribavirin is minimal. However, the evidence that T cells play a major role in treatment outcome is still fairly weak. Some studies have linked a boosting of T cell responses to sustained virologic responses [70], but others have found only limited evidence [71]. Treatment in acute disease, where therapy is most successful and T cells are at their highest frequency, is associated with, if anything, a reduction in T cell responses [72,73]. One plausible model is that T cell responses contribute to the long-term treatment outcome, but it is the pre-existing responses, rather than treatment-boosted responses, which are important. Under this model, if T cells and interferon are synergistic, then the markedly reduced T cell response to HCV in coinfection could still have a role to play in the poor treatment response.

### HIV therapy.

Emerging evidence suggests that long-term highly active ART does lead to a reduction in HCV-related liver disease [4,43–46]. Disease progression, which is affected by a number of other factors in such cohorts—including alcohol—is linked to low CD4^+^ T cell count, and reversal of this defect seems sufficient to reduce the rates of progression significantly.

What is not known is to what extent the treatment of HIV with effective ART can restore the T cell responses to HCV in the long term. Indeed, the clinical effect of ART on HCV load has been quite controversial, with small studies suggesting a range of different outcomes [74–77]. Certainly it does not appear a simple relationship whereby a drop in HCV load follows from a drop in HIV load, although this may occur rarely [78]. Restoration of CD4^+^ T cell responses against many other pathogens and antigens has been observed after ART, so might be expected in HCV. However, generally T cell responses against persistent pathogens such as CMV and Epstein-Barr virus are much stronger prior to immunosuppression than those against HCV [16]. If disease progression is slowed, without substantial increases in antiviral responses against HCV (linked to decreasing viral loads), this would support the idea that what is being repaired is not specific control over this tricky pathogen, but rather the immune dysregulation related to HIV replication. Further careful prospective studies are needed to address this critical point.

## Conclusions: Future Steps

The major impact of HIV on HCV infection has become clear in the last few years from careful large-scale clinical studies (Box 1). While a negative impact is to be expected from HIV coinfection, by comparison with other pathogens, the magnitude of the effect is actually striking.

Emerging evidence suggests that the major impact on virus load may well be explained by the observed changes in HCV-specific CD4^+^ and CD8^+^ T cell responses, particularly the striking changes in the former. However, this observation has shed important light on the substantial impact of these responses on the control of HCV in monoinfection, a feature which was perhaps underappreciated. The impact on disease progression may be influenced by high viral load, but it is very likely that defects in other regulatory populations play a role. In a similar way, defining these populations in more detail will give important clues into what controls the rate of progression in monoinfection. However, much more work needs to be done in this area.

Most importantly, the capacity to recover immunologically from this position is not yet established. There are numerous complex clinical considerations to take into account in deciding whom to treat and when, how and in what order, which are reviewed elsewhere [79]. The bottom line appears to be that effective therapy for HIV, in those with significant CD4 loss, has important additional benefits for HCV disease, even if HCV persists. Whether this effect occurs through restoration of HCV-specific immune responses or recovery of regulatory cell subsets or some other unrelated mechanism remains to be determined. Given their overall significance, at least defining the quality of immune responses and relating this to clinical parameters after the initiation of ART for HIV seems an important and practical goal.

HIV–HCV coinfection has created a significant clinical burden. Nonetheless, through the clinical insights gained it has provided some unique opportunities to understand the control of HCV and its pathogenesis. Hopefully in the future the growing scale of the problem will be matched by additional clinical–immunological studies to disentangle this further and potentially—in the longer term—provide further avenues for defining prognosis and improving treatment. 3

## Abbreviations

ART: antiretroviral therapy
CMV: cytomegalovirus
HCV: hepatitis C virus
HLA: human leukocyte antigen
IFN: interferon
IL: interleukin
TGF: transforming growth factor
